# Cognitive Impairment in Coeliac Disease with Respect to Disease Duration and Gluten-Free Diet Adherence: A Pilot Study

**DOI:** 10.3390/nu12072028

**Published:** 2020-07-08

**Authors:** Iain D Croall, Claire Tooth, Annalena Venneri, Charlotte Poyser, David S Sanders, Nigel Hoggard, Marios Hadjivassiliou

**Affiliations:** 1 Department of Infection, Immunity & Cardiovascular Disease, University of Sheffield, Sheffield S10 2JF, UK; n.hoggard@sheffield.ac.uk; 2 Institute for in silico Medicine, University of Sheffield, Sheffield S1 3JD, UK; 3 Department of Psychological Services, Royal Hallamshire Hospital, STH, Sheffield S10 2JF, UK; c.tooth1@nhs.net; 4 Department of Neuroscience, University of Sheffield, Sheffield S10 2HQ, UK; a.venneri@sheffield.ac.uk; 5 South West Yorkshire Partnership NHS Foundation Trust, Wakefield WF1 3SP, UK; charlotteapoyser@gmail.com; 6 Academic Unit of Gastroenterology, Royal Hallamshire Hospital, Sheffield Teaching Hospitals National Health Service Foundation Trust, Sheffield S10 2JF, UK; david.sanders1@nhs.net; 7 Department of Neurology, Royal Hallamshire Hospital, Sheffield Teaching Hospitals National Health Service Foundation Trust, Sheffield S10 2JF, UK

**Keywords:** coeliac disease, cognition, neurology, gluten-free diet, disease duration

## Abstract

Cognitive deficit has been reported in coeliac disease (CD), but previous reports often study heterogenous samples of patients at multiple stages of the disease, or lack control data. Healthy controls (*N* = 21), newly diagnosed CD patients (NCD; *N* = 19) and established CD patients (ECD; *N* = 35) were recruited from a specialist UK centre. Participants underwent a cognitive test battery that established seven overall domain scores. The SF-36 was administered as a quality of life (QoL) measure. Controlling for age, data were compared in between-group ANCOVAs with Tukey’s post-hoc test. Any significant outcome was compared in the ECD group only, between patients who were gluten-free diet adherent vs. non-adherent (defined via Biagi score and serology results). NCD and ECD groups underperformed relative to controls, by comparable degrees, in visual (overall model: *p* < 0.001) and verbal (*p* = 0.046) memory. The ECD group only underperformed in visuoconstructive abilities (*p* = 0.050). Regarding QoL, the NCD group reported lower vitality (*p* = 0.030), while the ECD group reported more bodily pain (*p* = 0.009). Comparisons based on dietary adherence were non-significant. These findings confirm cognitive deficit in CD. Dysfunction appears established at the point of diagnosis, after which it (predominantly) stabilises. While a beneficial effect of dietary treatment is therefore implied, future research is needed to establish to what extent any further decline is due to gluten exposure.

## 1. Introduction

Coeliac disease (CD) is an autoimmune disorder triggered by ingestion of gluten [1]. CD is a global problem, with a current estimated prevalence of 1% in most European countries [2]. Currently, the only treatment is strict adherence to a gluten-free diet.

Patients suffering with CD can have an abnormal reaction to gluten that classically causes gastrointestinal symptoms such as abdominal pain, diarrhoea, constipation, bloating and weight loss (sometimes referred to as classic CD). Yet some patients may not present with any gastrointestinal symptoms but may have extraintestinal manifestations. It is now accepted that CD is a multi-system disorder and can be associated with anaemia, osteoporosis, liver disease and skin manifestations [3,4]. Furthermore, there is increasing evidence that CD can present with neurological problems such as cerebellar ataxia, peripheral neuropathy and encephalopathy [5].

Cognitive deficits are often reported by patients with CD. CD has been associated with ‘brain fog,’ which refers to a range of symptoms including cognitive slowing, difficulty concentrating and problems with short- and long-term memory. One study found that patients with gluten ataxia showed significant verbal memory deficits [6]. Recent recognition of the role of the cerebellum in cognitive functions (cerebellar cognitive affective syndrome) could potentially explain some of these deficits, given that the cerebellum is very frequently the target organ in the context of CD [7]. Furthermore, gluten encephalopathy encompasses patients with such cognitive deficits, sometimes associated with frequent headaches and an abnormal MRI scan showing excessive white matter changes over and above those associated with aging [8]. It is currently unclear what the clinical implications of these brain abnormalities are for CD patients. A large epidemiological study found that patients with CD were at an increased risk of vascular dementia [9]. A possible relationship between early onset dementia and CD has also been identified, with patients showing moderate to severe intellectual deterioration. Diffuse cerebral or cerebellar atrophy was also detected on CT scans [10].

An early study by Hallert and Astrom [11] investigated a series of newly diagnosed, consecutive CD patients and found borderline cognitive impairment in 21% of cases. Contemporary research investigating this is relatively scarce but has continued to produce positive findings. One study found that CD patients who had been treated for an average of 5.5 years performed worse than controls on tests examining executive functioning and processing speed [12]. Another has indicated that some cognitive functions, also including visual memory, improve in patients after consuming a gluten-free diet (GFD) for a year [13]. Finally, a recent study confirmed the presence of a reaction time deficit compared to controls in a cohort of CD patients taken from a national UK database [14]. However, findings such as these have also been refuted by a study that found no evidence of cognitive impairment in a group of 28 newly diagnosed CD patients [15]. It should be noted that this study potentially lacked experimental sensitivity, as it only used a very brief screening measure of cognitive functioning. Furthermore, they compared the performance of CD patients with a control group of patients who presented to their gastroenterology clinic with Coeliac-like symptoms but in whom CD was ruled out. The authors did not comment on the aetiology of the symptomatic control group and they did not compare performance with a healthy control sample. As such, the conclusion of no cognitive impairment is questionable.

Another limitation of such studies that look at cognitive dysfunction in patients with established CD is that very few control for the effect of disease stage/treatment. The one aforementioned study which examined cognitive change over time from the point of diagnosis indicated a degree of recovery [13], suggesting this to be an important variable to consider. A GFD has otherwise been established as beneficial for patients with gluten ataxia, gluten encephalopathy and gluten neuropathy [16]. Therefore, it is imperative that future studies on patients with CD stratify based on whether the condition is newly diagnosed or established. Further, no study has both examined cognitive change over time and simultaneously included control data to ascertain how any scores deviate from a baseline level of expected performance.

To further understand the prevalence of cognitive problems in CD and the impact of a gluten-free diet, the current study collected pilot data on patients with CD at different disease stages to address the following three main questions:

(1) Do newly diagnosed patients with CD have cognitive difficulties when compared to healthy controls?
(2) Do patients with a longstanding diagnosis of CD differ from newly diagnosed patients with regard to their cognitive profile?
(3) Do patients who comply with a gluten-free diet have better cognition than those who do not?

## 2. Materials and Methods

### 2.1. Participants

CD participants were recruited from a specialist coeliac disease clinic held at Sheffield Teaching Hospitals NHS Trust from September 2015 to July 2016. To be eligible for this study, all participants had to be aged between 18 and 70 years and be proficient in English language. It was important to include a wide age range, as CD can be diagnosed at any age [17]. To be included in the CD groups, their diagnosis had to be based on a small bowel biopsy histology in keeping with CD (triad of villus atrophy, crypt hyperplasia and increased intraepithelial lymphocytes) taken at baseline.

Patients with newly diagnosed CD were approached consecutively during this period when they attended their clinic appointment post-biopsy result. A second group of patients with established CD (had held their diagnosis for at least 5 years) were identified from a database at the same clinic and invited to take part by the study team. This established group was further separated into those who were gluten-free diet adherent and those who were not. This was determined by the participant’s responses on the Biagi scale [18] and their last clinical serological analysis for gliadin antibodies (which they receive as part of routine clinical care; ELISA assay, Phadia 2500) in combination with the local clinical cut-offs. Being adherent to the diet was defined as being both adherent according to Biagi score and serological negativity.

The control participants were a convenience sample including friends and family of patients attending the clinic. These subjects did not have a diagnosis of CD or gluten sensitivity and were not following a GFD. The project investigators intended to match the control group with the CD patients as closely as possible in terms of age, gender, years of education and estimated pre-morbid functioning. Participants in all groups were excluded if they had any known neurological conditions, psychiatric conditions and a history of substance abuse. Participation in this study was voluntary. However, travel expenses were available. This study was approved by the South West Cornwall and Plymouth Regional Ethics Committee (REC reference; 15/SW/0096, 10/04/2015). All participants provided written informed consent.

**Figure 1:**
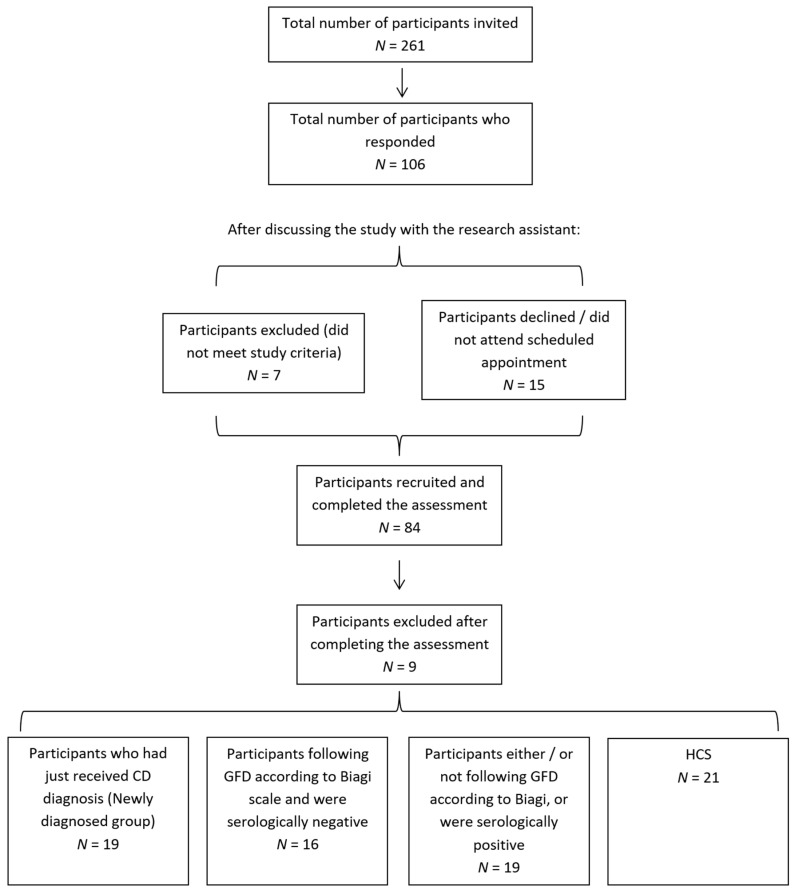
Participant recruitment process, including sample sizes. HCS, healthy control subjects.

The overall patient recruitment process is visualised in Figure 1. The healthy controls (*N* = 21) had a mean(SD) age of 43.5 (16.2), and were 76.2% female. The newly diagnosed CD cases (*N* = 19) were aged 45.1 (17.3) and were 84.2% female. The overall established CD group (*N* = 35) had a mean(SD) age of 55.5 (12.7), were 88.6% female and were a mean of 11.8 years post-diagnosis (range: 5.2–45.1, SD = 7.8). Of these, 16 were determined to be dietary adherent while 19 were not.

### 2.2. Study Power

This analysis is presented as a pilot study. Nonetheless, previous comparable investigations of cognitive outcomes in CD have used sample sizes which are smaller than those in the current analysis. Casella et al. [12] used two groups of 18, while Lichtwark et al. [13] used a repeated-measures design on a single group of 11. Each of these papers reported significant findings in outcomes from cognitive testing, indicating that they were sufficiently powered to detect experimental effects.

### 2.3. Design

This study followed a cross-sectional design to confirm or reject the presence of cognitive deficits at different stages of CD.

### 2.4. Assessment Procedure

Participants attended one appointment for 2 h with the research assistant where written consent was provided. The newly CD diagnosed participants had to be tested within 4 weeks of receiving their diagnosis. All participants completed the same neuropsychological assessments in a consistent order to ensure that the delay conditions were adhered to. All of the assessments consisted of standardised clinical instruments, administered according to the standardised instructions provided by the assessment manuals. Quality of life measures were also included to investigate the relationship between gluten-free diet adherence, symptomatology and cognitive difficulties. Any participant who did not complete all outcomes was excluded from analysis.

### 2.5. Testing Battery and Initial Data Handling

The cognitive test battery included (1) the Test of Premorbid Functioning (ToPF); (2) the Wechsler Adult Intelligence Scale-III (WAIS) tests of block design, vocabulary, matrix reasoning and similarities; (3) Trail Making Test (TMT); (4) Controlled Oral Word Association Test (letter fluency only, COWAT); (5) Digit Span; (6) story recall; (7) California Verbal Learning Test (CVLT); (8) Rey–Osterrieth Complex Figure Test (CFT); (9) Digit-Symbol Coding; (10) Speed of Information Processing (SoIP); (11) Boston Diagnostic Aphasia Examination for Verbal Agility (BDAE Verbal Agility) [19].

Key scores for each test were identified according to common convention. On examination, it was found that the BDAE Verbal Agility total score exhibited a strong ceiling effect, wherein the vast majority of participants scored maximum points. It was therefore decided to ignore this outcome in the main analyses to maintain experimental sensitivity. The ToPF IQ was calculated so that experimental groups could be compared on this. Otherwise, all cognitive variables were converted to Z scores relative to the performance mean and standard deviation of the control group. It was further ensured that all outcomes were transformed (if needed) such that a higher score always represented a better performance.

Specific outcomes were then averaged, with equal weighting, into seven overall cognitive “domain” scores. Their names and composition were as follows: mental flexibility—raw scores from WAIS block design and matrix reasoning, TMT (“B” condition as a percentage of “A” condition) and COWAT letter fluency; visuoconstructive abilities—WAIS block design raw score and CFT copy condition; verbal ability—raw scores from WAIS vocabulary and similarities; overall verbal memory—immediate and delayed scores from story recall, and from the CVLT immediate free recall, delayed free recall, recognition “hits” and recognition “false positives”; overall visual memory—immediate and delayed scores from the CFT; working memory—Digit Span backwards; processing speed—TMT (“A” condition), Digit-Symbol Coding raw score and SoIP motor-adjusted score.

Subdomains were also constructed for overall verbal memory and overall visual memory, such that short-term (ST) and long-term (LT) components of these may be separately examined (e.g., LT verbal memory would specifically include “delayed” verbal memory tasks and the “recognition” portion of the CVLT).

For quality of life (QoL), the SF-36 was used [20]. Raw scores from this were converted to the recommended eight outcomes for physical functioning, physical roles, bodily pain, vitality, general health, social functioning, emotional roles and mental health.

### 2.6. Statistical Analysis

Analyses were performed using SPSS Version 25.

All variables were visually inspected for normality to determine what form of testing should be used. A non-normal distribution would mean that the appropriate non-parametric test would be used in cases examining only a single outcome. However, for analyses with an additional controlling covariate (e.g., age), an ANCOVA was performed and the distribution of residuals/error were inspected for normality to confirm that the model assumptions had not been broken.

Major groups (controls/newly diagnosed cases/all established cases) were statistically compared on age, sex, years of education and ToPF IQ (univariate ANOVAs/chi-squared analyses). Any variable where significant differences were found would be used as a covariate in relevant analyses. Established CD subgroups (i.e., based on dietary adherence) were also compared on these variables, which would then be used as a covariates in the same manner.

For all cognitive domain and QoL outcomes, the three major groups were compared by univariate ANOVA. Significant findings from these initial analyses would justify further post-hoc testing. Tukey’s post-hoc test was used to confirm the specific groups where the differences lay. Further, if differences were found in either overall visual/verbal memory cognitive domains, then the ST and LT subdomains of these were also compared in the same manner, with additional testing being conducted as appropriate to determine whether a LT deficit was unique, or a consequence of an accompanying ST deficit.

Established CD dietary adherence subgroups were compared to one another on any variable where a significant difference was reported in these primary comparisons.

Finally, if justified by other findings, correlations between significant cognitive and QoL outcomes would be considered.

## 3. Results

### 3.1. Analyses of Samples’ Demographics

Groups were significantly different for age (ANOVA; *p* = 0.007), wherein the established CD cases were older than both newly diagnosed cases (*p* = 0.044) and controls (*p* = 0.013), who were not themselves different from one another. Otherwise there were no significant differences for any other “demographic” variable, including ToPF IQ. Established CD subgroups based on dietary adherence were also not significantly different from one another on any of these variables, or the number of years since their diagnosis. Full details are available in Table 1.

### 3.2. Analyses of Cognitive Performance

**Figure 2:**
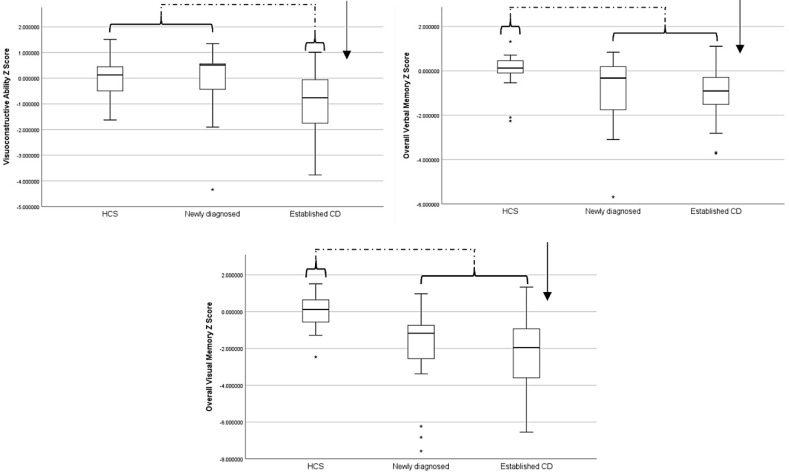
Boxplots visualising group performance on cognitive domains found to be significantly different in statistical analyses. Asterisks represent data points identified as outliers by the statistical software. Brackets and arrows are superimposed to demonstrate the significant difference between groups, with reference to control group performance, within each model. HCS, healthy control subjects.

ANCOVAs controlling for age revealed that groups were significantly different from one another in visuoconstructive abilities (*p* = 0.050), overall verbal memory (*p* = 0.046) and overall visual memory (*p* < 0.001) domains. Tukey’s post-hoc test determined that in the case of visuoconstructive ability, the group difference was driven by established CD cases performing significantly worse than both newly diagnosed cases and controls (who were not themselves different from one another). However, in the case of both overall visual and verbal memory domains, the significant effect was driven by established and newly diagnosed groups performing significantly worse than controls (while not themselves being different from one another). Full details are presented in Table 2 and Figure 2.

Further analysis on the long-term (LT) and short-term (ST) components of the verbal and visual memory domains revealed that the verbal memory finding had been driven by a worsening of the LT memory subcomponent specifically (*p* = 0.019), and that the ST memory subcomponent did not exhibit any significant effects (Table 3). However, both ST (*p* < 0.001) and LT (*p* = 0.002) visual memory subcomponents had worsened in CD cases. Tukey’s post-hoc test confirmed that the between-group pattern of findings was not different in these subdomains compared to the parent analyses. A further ANCOVA was conducted to control for ST visual performance in explaining the LT visual outcome (i.e., ST domain scores were included as a covariate in addition to age and group). Here, the study group did not retain its significance (*p* = 0.631), while ST performance was highly significant (*p* < 0.001).

In the analysis of the SF-36 scores, significant effects were observed for items assessing bodily pain (*p* = 0.009) and vitality (*p* = 0.030, Table 4). Tukey’s post-hoc test found that the bodily pain result was driven by established CD cases (44.14 ± 11.56) having a worse outcome than controls (54.39 ± 10.51). However, the vitality result was driven by newly diagnosed CD cases (47.83 ± 10.24) having a worse outcome than controls (55.16 ± 5.81).

Testing was performed to compare dietary adherence subgroups within the established CD cases on all variables where a significant effect was found in the major group analyses as described above. However, no significant subgroup differences emerged.

Correlation analyses were conducted separately within each experimental group for a provisional investigation of any relationships between cognitive and QoL variables which had been found to be significant in the above analyses. However, no significant effects were observed. Finally, an alternative means of controlling for age differences between major groups was implemented. Significant between-group findings (i.e., verbal/visual memory, and visuoconstructive ability) were re-analysed via independent samples *t*-tests using subsamples of the control (now *N* = 10, age = 55.4) and newly diagnosed (now *N* = 12, age = 55.7) groups, which were age-matched against the original, established CD group. These analyses all retained significance and the original pattern of difference.

## 4. Discussion

In this study, the impact of CD on cognitive performance and QoL was assessed with respect to duration of illness and dietary success. Both newly diagnosed CD and established CD patients were found to underperform compared to controls in tests investigating verbal and visual memory. Established CD cases were also found to underperform compared to both newly diagnosed cases and controls in measures of visuoconstructive ability. Further, worse QoL outcomes were variably observed between patient groups, with newly diagnosed patients specifically reporting lower vitality and established patients specifically reporting worse bodily pain. Comparisons of these preliminarily significant variables in subgroups of established patients based on their dietary adherence did not reveal any significant results. In addition, no significant correlation was found between cognitive scores and QoL measures.

Cognitive deficit in CD has been established in limited previous studies [11,12,13,14]. In addition to evidence of amnesia, acalculia, confusion and personality change, the cognitive domains reported to be affected from these include components of executive functioning, processing speed and memory. Other case and small group studies on patients with CD presenting with cognitive dysfunction have shown variable responses to a GFD [10,21,22], although these relied on clinical cognitive assessment reports, which may be relatively insensitive at detection of more “subtle” forms of deficit [23]. Overall, this past research has lacked investigations which simultaneously consider the potential beneficial effect of a GFD over time, and which include control data to establish a meaningful performance baseline against.

The primary analyses of the current study considered cognitive performance between a control group and two groups of CD patients based on the stage of their disease—newly diagnosed or established. In these comparisons, CD groups were observed to underperform in three of the seven domains investigated. For visual and verbal memory domains, post-hoc testing indicated that the deficit was present in the newly diagnosed patients and had not appeared to worsen further when compared to established patients. Further analysis of these data found that these findings were driven by a long-term memory deficit in both domains, but a short-term deficit in only visual memory. Additional investigation of the visual memory outcomes confirmed that the underperformance in the long-term tests was likely driven by the accompanying short-term deficit rather than being a separate and unique consequence. CD patients also underperformed in tests of visuoconstructive abilities—except scores on this domain were comparable between controls and the newly diagnosed group, with the established cases specifically demonstrating a deficit. Taken together, these results demonstrate that cognitive deficit may be present at the earliest point of diagnosis but appears predominantly stable following treatment. The exception to this is the finding concerning visuoconstructive ability, which indicates that a degree of further decline may still be expected.

The affected domains in the current study show some agreement with previous literature; for visual memory, the Complex Figure Test was used, which was the same task that Lichtwark et al. observed patients with CD to improve on, following a year of a GFD [13]. However, while problems concerning mental flexibility and processing speed have been reported [12,13,14], these appear preserved in the current analysis. This disparity may be due in part to some of the specific tasks used differing between studies or issues of study power. Identification of a set of cognitive markers which are dependably affected in CD is highly desirable, and more research is required for this.

Circulating antibodies against gluten products are thought to be the primary cause of underlying brain pathology in gluten-related conditions. Transglutaminase 6 (TG6), an antibody common in gluten ataxia and also present in CD patients at a rate of approximately 40% [24], is expressed in a number of brain regions and particularly the cerebellum [25]. This may explain the characteristic patchy loss of cerebellar Purkinje cells observed in gluten ataxia [7]. Antibodies against gliadin are ubiquitously produced in CD and have also been seen to exhibit reactivity with blood vessels which supply the brain [26]. This in turn may hypothetically lead to disruption of the blood–brain barrier and further downstream consequences such as vascular-driven white matter injury, which CD patients are increasingly seen to be at risk of [9]. CD also involves many nutritional consequences, and treated patients are at a raised risk of micronutrient deficiencies [27]. Some research has linked low levels of, e.g., folic acid [28] and vitamin D [29] to cognitive deficit. However, as routine nutritional monitoring is recommended by NICE guidelines for CD, it is unlikely that this would contribute to the findings of the current study.

Imaging studies of CD have been integral in characterising the brain tissue injury found in CD. Lowered brain volume in the cerebellum (whole cerebellar grey matter) and thalamus has been found in patients with TG6 positivity [24], while white matter changes affecting the tract connecting those regions (spinothalamic) in addition to the corpus callosum have been reported as a more general finding [14]. These brain locations are known to hold associations with the cognitive functions found to be impaired in the current study. The cerebellum is increasingly becoming recognised as playing an integral role in a range of cognitive functions, including ones underlying verbal and visual memory [30]. The thalamus is also reported to hold a number of associations with types of memory [31].

The finding of cognitive deficit in newly diagnosed patients agrees with previous research showing other neurological symptoms to be prevalent at the same stage in CD [24]. The pattern of our current results showing cognitive scores are often stable between new and established CD cases is also in general agreement with previous literature that a GFD is beneficial in preventing further neurological decline [13,16]. However, additional testing of established patient subgroups based on markers of their dietary adherence did not reveal any significant findings. These subgroups were defined by both their Biagi score and serological test results. Reliable assessment of GFD adherence is difficult, and measures of it (including those used in the current study) have been shown as lacking sensitivity [32]. It is possible that this issue, in combination with the diminished sample size for these analyses, contributed to a lack of findings. Dietary success is the foundation of CD treatment, and further cognitive research utilising more accurate methods of tracking is needed.

Quality of life was also examined, assessed by the SF-36 questionnaire. Significant findings here differed based on disease duration. Vitality was seen to be impacted in the newly diagnosed group. This is not necessarily surprising, given that this essentially describes energy/fatigue, which may still be impacted due to patients only recently having adopted a GFD. However, bodily pain was seen to be worsened in established patients only. While some research has shown that health-related QoL in CD is primarily dependent on dietary adherence [33], others have demonstrated the relationship may be more complicated and dependent on perceived, rather than actual, success [34]. Studies have also reported that sleep disorders are common in newly diagnosed CD patients and do not improve 1 year after following a GFD, and that this has a connection to depression, anxiety and fatigue [35]. In the current study, further analysis of the bodily pain result based on dietary compliance groups showed no significant findings. Due to the aforementioned issues of sensitivity in these groups, it should not be ruled out that this may still be due in part to, e.g., abdominal pain caused by ongoing gluten exposures among some of the patients. However, this also potentially supports such previous research showing that diminished QoL in CD is an ongoing problem that is relatively detached from engagement with a GFD.

This study had some limitations. The sample size, while comparable to other research investigating cognitive outcomes in CD [12,13], is relatively small. It is for this reason that we present the findings as pilot analyses and highlight that future research should be conducted with larger cohorts to increase power. Doing so may increase the sensitivity of analyses such as those based on markers of dietary adherence to detecting further experimental effects. The study groups also had large age ranges, introducing heterogeneity into the data. Age is highly relevant to performance on cognitive tests [36], and although this was controlled for in the analyses, it is possible that this nonetheless impacted analyses to an extent. Future research should use more tightly defined experimental groups. Finally, a cut-off of 4 weeks post-diagnosis was used for the newly diagnosed CD group criteria. This was to ensure that no participant would have engaged with a GFD for a significant length of time, although some heterogeneity in possible treatment effects may still be present within this timeframe.

## 5. Conclusions

In summary, in this study, we have demonstrated cognitive deficit in CD revolving around verbal memory, visual memory and visuoconstructive abilities. Stratifying by disease duration, the pattern of these findings suggests that cognitive deficit is established at the point of diagnosis, and though it remains generally stable after this point, some further decline may be expected. QoL was also observed to be impacted in established CD with respect to bodily pain. The extent to which these problems are due to issues around dietary compliance remains an open and important question for further research.

## Figures and Tables

**Table 1:** Demographics of the study groups.

Variable	HCS	Newly Diagnosed	All Established Cases	Results of Statistical Comparison between Major Groups	Subgroup: Established CD and Diet Adherent	Subgroup: Established Case and Not Diet Adherent	Results of Test Comparing Established CD Subgroups
Sample Size	21	19	35	-	16	19	-
Age	43.5 ± 16.2	45.1 ± 17.3	55.5 ± 12.7	***p* = 0.007 ^1^** F = 5.329	57.8 ± 10.6	53.6 ± 14.3	*p* = 0.481
% Female	76.2% (*N* = 16)	84.2% (*N* = 16)	88.6 (*N* = 31)	*p* = 0.473	93.8% (*N* = 15)	84.2% (*N* = 16)	*p* = 0.377
Years of Education	15.2 ± 2.0	14.1 ± 3.0	13.8 ± 3.0	*p* = 0.172 F = 1.802	14.2 ± 3.2	13.4 ± 2.8	*p* = 0.452
ToPF IQ	106.4 ± 5.6	104.4 ± 8.8	104.8 ± 8.2	*p* = 0.662 F = 0.415	106.6 ± 6.8	103.3 ± 9.1	*p* = 0.239
Years since coeliac diagnosis	-	0.0	11.8 ± 7.8	-	11.4 ± 4.4	12.1 ± 9.8	*p* = 0.803

^1^ Established CD cases were significantly older than both the newly diagnosed group (*p* = 0.044) and controls (*p* = 0.013). Data are the means and standard deviations. HCS, healthy control subjects.

**Table 2:** Results of groups comparisons (ANCOVAs, controlling for age) on the cognitive domain scores.

Cognitive Outcome	HCS Mean ± SD	Newly Diagnosed Mean ± SD	Established CD Mean ± SD	*p* Value F Value
**Mental Flexibility**	0.00 ± 0.60	−0.14 ± 1.05	−0.80 ± 0.95	*p* = 0.064 F = 2.850
**Visuoconstructive Ability**	0.00 ± 0.79	−0.11 ± 1.38	−0.91 ± 1.09	***p* = 0.050 ^1^** F = 3.130
**Verbal Ability**	0.00 ± 0.87	−0.07 ± 1.67	−0.59 ± 1.03	*p* = 0.129 F = 2.105
**Overall Verbal Memory**	0.00 ±0.83	−0.97 ± 1.65	−0.93 ± 1.19	***p* = 0.046 ^2^** F = 3.216
**Overall Visual Memory**	0.00 ± 0.96	−2.04 ± 2.40	−2.49 ± 2.02	***p* < 0.001 ^3^** F = 8.517
**Working Memory**	0.00 ± 1.02	−0.26 ± 1.51	−0.18 ± 1.66	*p* = 0.762 F = 0.273
**Processing Speed**	0.00 ± 0.74	0.31 ± 1.02	−0.13 ± 1.03	*p* = 0.332 F = 1.121

^1^ For visuoconstructive ability, established CD cases performed worse than newly diagnosed (*p* = 0.034) and control groups (*p* = 0.010). ^2^ For overall verbal memory, established (*p* = 0.021) and newly diagnosed (*p* = 0.042) groups performed worse than controls. ^3^ For overall visual memory, established (*p* < 0.001) and newly diagnosed (*p* = 0.003) groups performed worse than controls. HCS, healthy control subjects.

**Table 3:** Results of additional analyses comparing short-term (ST) and long-term (LT) components of visual and verbal memory between groups.

Cognitive Outcome	HCS Mean ± SD	Newly Diagnosed Mean ± SD	Established CD Mean ± SD	*p* Value F Value
**ST Verbal Memory**	0.00 ± 0.59	−0.29 ± 0.80	−0.29 ± 0.74	*p* = 0.478 F = 0.745
**LT Verbal Memory**	0.00 ± 0.85	−1.23 ± 1.91	−1.18 ± 1.34	***p* = 0.019** F = 4.206
**ST Visual Memory**	0.00 ± 1.02	−2.13 ± 2.31	−2.65 ± 2.09	***p* < 0.001** F = 9.472
**LT Visual Memory**	0.00 ± 1.02	−1.95 ± 2.51	−2.33 ± 2.01	***p* = 0.002** F = 7.089

Tukey’s post-hoc test confirmed that the pattern of between-group differences replicated the corresponding analyses concerning overall visual and verbal memory domains. HCS, healthy control subjects.

**Table 4:** Results of “major group” ANCOVA analyses (controlling for age) comparing outcomes from the SF-36 between controls, newly diagnosed CD cases and established CD cases.

SF-36 Outcome	HCS Mean ± SD	Newly Diagnosed Mean ± SD	Established CD Mean ± SD	Model Results
**Physical Functioning**	51.33 ± 10.21	51.50 ± 8.24	47.49 ± 10.18	*p* = 0.732 F = 0.313
**Physical Role**	52.85 ± 7.26	50.64 ± 10.40	46.93 ± 10.10	*p* = 0.204 F = 1.627
**Bodily Pain**	54.39 ± 10.51	50.82 ± 9.06	44.14 ± 11.56	***p* = 0.009** F = 4.977
**General Health**	50.51 ± 6.21	46.91 ± 9.01	44.80 ± 10.33	*p* = 0.127 F = 2.126
**Vitality**	55.16 ± 5.81	47.83 ± 10.24	51.10 ± 8.67	***p* = 0.030** F = 3.700
**Social Functioning**	54.27 ± 4.71	48.85 ± 7.83	49.06 ± 9.60	*p* = 0.094 F = 2.449
**Emotional Role**	51.80 ± 9.01	48.66 ± 11.21	51.10 ± 8.14	*p* = 0.534 F = 0.632
**Mental Health**	50.44 ± 6.89	49.14 ± 10.37	50.90 ± 9.69	*p* = 0.841 F = 0.173

Significant differences were found in bodily pain, where established CD cases have worse scores than HCS (*p* = 0.002), and vitality, where newly diagnosed cases have worse scores than HCS (*p* = 0.021). HCS, healthy control subjects.
